# Correction: Takayama et al. Polyvinyl Butyral Addition Effects on Notched Charpy Impact Strength of Injection-Molded Glass Fiber-Reinforced Polypropylene. *Polymers* 2024, *16*, 3472

**DOI:** 10.3390/polym17070916

**Published:** 2025-03-28

**Authors:** Tetsuo Takayama, Yuuki Yuasa, Quan Jiang

**Affiliations:** Graduate School of Organic Materials Science, Yamagata University, Yamagata 990-8510, Japan

## Table and Figure Corrections

There was an error in the original publication [1]. There was an error in the description of Table 2, Figures 14 and 15 and Equation (13). Specifically, the error in the fiber length measurement method was rectified. The corrected Table 2, Figure 14 and Figure 15 and Equation (13) appear below.

(13) $$a_{iN}=\left\{\begin{array}{c} \frac{\tau_{i}V_{f}L_{F}^{2}}{d}\cos\varphi \mathrm{for}\;L_{F}<L_{c}\\ \frac{dV_{f}\sigma_{FB}^{2}}{4\tau_{i}}\cos\varphi \mathrm{for}\;L_{F}>L_{c} \end{array}\right.$$

The authors state that the scientific conclusions are unaffected. This correction was approved by the Academic Editor. The original publication has also been updated.

## Figures and Tables

**Figure 14:**
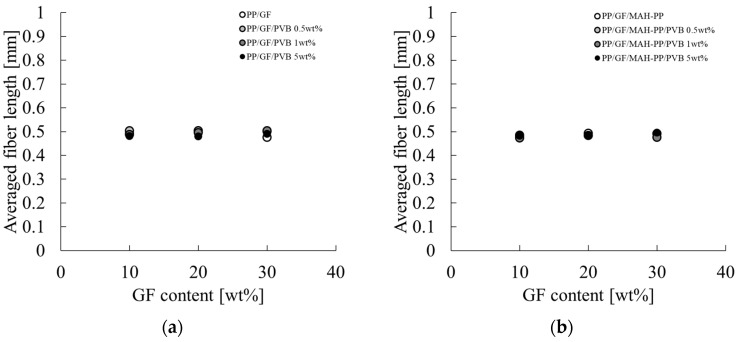
GF content dependences on the averaged fiber length of (**a**) PP/GF/PVB and (**b**) PP/GF/MAH-PP/PVB composites.

**Figure 15:**
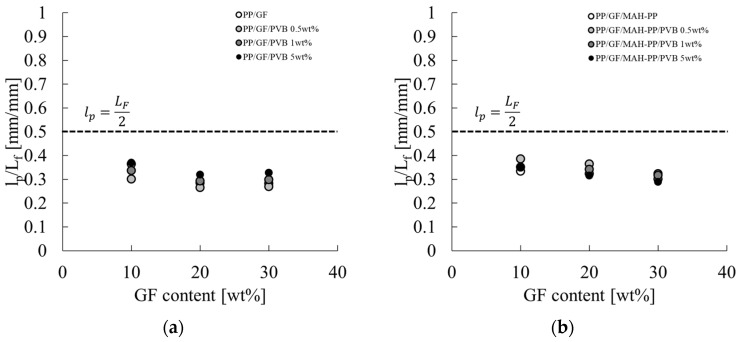
GF content dependences on the l_p_/L_F_ of (**a**) PP/GF/PVB and (**b**) PP/GF/MAH-PP/PVB composites.

**Table 2:** Characteristic values used for this study.

PP (wt%)	GF (wt%)	MAH-PP (wt%)	PVB (wt%)	IFSS (MPa)	σ_w_ (MPa)	a_iN_ (kJ/m^2^)	γ_i_ (N/mm)	φ (degree)	L_f_ (mm)	σ_FB_ (MPa)	σ_FB0_ (MPa)
90	10	0	0	7.6	22.7	2.7	0.99	68.9	0.49	419	1928
80	20	0	0	7.4	21.4	3.5	0.43	63.9	0.50	326	1618
70	30	0	0	6.3	18.0	3.9	0.20	57.8	0.48	261	1460
89.5	10	0	0.5	8.4	22.4	2.1	0.65	69.2	0.50	389	1782
79.5	20	0	0.5	7.7	20.1	3.2	0.19	66.1	0.50	317	1520
69.5	30	0	0.5	6.9	17.4	4.2	0.04	56.3	0.50	286	1653
89	10	0	1	8.5	23.4	2.5	1.01	68.6	0.49	429	1981
79	20	0	1	7.3	20.2	3.3	0.33	59.0	0.50	325	1772
69	30	0	1	6.1	17.7	4.4	0.14	51.4	0.50	281	1843
85	10	0	5	7.2	20.3	2.5	1.77	71.4	0.48	391	1744
75	20	0	5	7.2	18.4	3.9	0.82	63.2	0.48	340	1706
65	30	0	5	5.7	14.6	5.0	0.48	56.2	0.49	281	1625
85	10	5	0	8.9	25.9	2.6	1.22	71.2	0.49	443	1975
75	20	5	0	8.5	25.3	5.0	0.57	66.5	0.49	418	1990
65	30	5	0	8.5	22.9	6.7	0.31	62.0	0.49	387	1987
84.5	10	5	0.5	8.2	24.8	3.1	0.99	72.2	0.48	468	2066
74.5	20	5	0.5	7.9	24.0	5.6	0.41	65.8	0.48	425	2044
64.5	30	5	0.5	7.8	22.2	6.9	0.20	59.6	0.49	383	2063
84	10	5	1	8.3	26.5	2.4	1.31	63.7	0.47	423	2106
74	20	5	1	8.0	25.6	4.8	0.57	61.1	0.48	406	2119
64	30	5	1	7.7	23.6	5.9	0.30	56.2	0.48	358	2077
80	10	5	5	8.0	25.2	2.5	2.13	69.4	0.48	412	1881
70	20	5	5	7.9	24.4	4.3	1.09	66.0	0.48	372	1780
60	30	5	5	7.8	23.0	5.8	0.71	61.9	0.50	342	1758
